# Time window for early intervention in tauopathy mouse model

**DOI:** 10.1002/alz.088882

**Published:** 2025-01-09

**Authors:** Benito Maffei, Nathaneal O'Neill, Chiara Dyson, Guru Padmasola, Francisco Moreira, Marco Leite, Vincent Magloire, Dimitri Michael Kullmann, Gabriele Lignani

**Affiliations:** ^1^ UCL, London UK

## Abstract

**Background:**

Early changes in network activity serve as distinctive hallmarks in tauopathies such as Alzheimer’s Disease and Frontotemporal Dementia. Decades before the onset of cognitive decline, many patients experience seizures which exacerbate the condition. Despite the recognised etiological overlaps between tauopathies and epilepsies, the relationship between tau and the development of circuit hyperexcitability is unclear and needs further investigation.

**Method:**

Using wireless EEG to record intrahippocampal local field potentials (LFP), we characterised early biophysical alterations within the hippocampus of tauopathy mice.

**Result:**

We identified a novel transient time window where hippocampal function is disrupted before the onset of tau pathology and cognitive decline

**Conclusion:**

A precise and robust targeting of this circuit prior to the manifestation of the disease would advance our understanding of the role of circuit excitability in tau pathology. This could serve as an early therapeutic target, offering a novel strategic approach to deter the progression of the disease.

